# The rationale for development of ligelizumab in food allergy

**DOI:** 10.1016/j.waojou.2022.100690

**Published:** 2022-09-13

**Authors:** Robert A. Wood, R. Sharon Chinthrajah, Alexander Eggel, Ivan Bottoli, Aurelie Gautier, Maximilian Woisetschlaeger, Paolo Tassinari, Pablo Altman

**Affiliations:** a Division of Allergy & Immunology, Department of Pediatrics, Johns Hopkins University School of Medicine, Baltimore, MD, USA; b Sean N. Parker Center for Allergy and Asthma Research, Stanford University, Stanford, CA, USA; c Department of BioMedical Research, University of Bern, Bern, Switzerland; d Department of Rheumatology and Immunology, University Hospital Bern, Bern, Switzerland; e Novartis Pharma AG, Basel, Switzerland; f Novartis Pharmaceuticals Corporation, East Hanover, NJ, USA

**Keywords:** Food allergy, IgE, Ligelizumab, Omalizumab, Talizumab

## Abstract

Food allergy (FA) is a growing healthcare problem worldwide and the rising prevalence in many countries can be attributed to lifestyle, environmental, and nutritional changes. Immunoglobulin E (IgE)-mediated FA is the most common form of FA affecting approximately 3%–10% of adults and 8% of children across the globe. Food allergen–induced immediate hypersensitivity reactions mediated by IgE and high-affinity IgE receptor (FcεRI) complexes on mast cells and basophils are a major hallmark of the disease. FA can affect several aspects of health-related quality of life and impose a substantial financial burden on patients and healthcare systems. Although currently there is one United States Food and Drug Administration (FDA) and European Medicines Agency (EMA)–approved treatment for peanut allergy (Palforzia), the main treatment approaches are based on allergen avoidance and symptom management. Thus, there is an urgent need for more effective and ideally disease-modifying strategies. Given the crucial role of IgE in FA, anti-IgE monoclonal antibodies are considered promising therapeutic agents. Talizumab was the first humanized anti-IgE antibody to demonstrate substantial protection against allergic reactions from accidental peanut exposure by substantially increasing the peanut reactivity threshold on oral food challenge. However, development of talizumab was discontinued and further trials were performed using omalizumab. In double-blind, Phase 2, placebo-controlled trials in patients with multi-FAs, sustained dosing with omalizumab, or omalizumab in combination with oral immunotherapy, enabled rapid desensitization to multiple trigger foods. In this review, we describe the development of ligelizumab (a derivative of talizumab), a next generation, humanized monoclonal anti-IgE antibody, its existing clinical evidence, and its potential in the management of FA. When compared with omalizumab, ligelizumab binds with ∼88-fold higher affinity for human IgE and recognizes a different epitope that substantially overlaps with the binding site of FcεRI. These properties translate into a high potency to block IgE/FcεRI signaling in both *in vitro* and *in vivo* studies. Given its efficient suppression of IgE levels, good safety and pharmacokinetic/pharmacodynamic profile, ligelizumab clearly warrants further studies for the potential management of FA.

## Introduction

Food allergy (FA) refers to the reproducible adverse immune response to antigens delivered orally and is characterized by the breakdown of mucosal immune tolerance against certain ingested foods (eg, milk, egg, peanut).^1^^,^^2^ The prevalence of FA has been rapidly and increasing in children over the past couple of decades, and although data regarding changes in the prevalence in adults are more limited, FA is common at all ages.^3^ Indeed, an increased incidence of new-onset FA has been reported in adults, with wheat, shellfish, soy, tree nut, and fin fish FAs being the most common adult-onset FAs.^4^ A rapidly rising prevalence of FA in many countries (eg, United States, United Kingdom, Australia, China, and Taiwan) suggests that it is an emerging health priority, particularly in the more economically developed countries.^5^, ^6^, ^7^

Compared with other classic allergic conditions such as allergic rhinitis, allergic asthma, atopic dermatitis, and non-classic conditions such as nasal polyposis and chronic spontaneous urticaria (CSU), FA seems to have a stronger association with immunoglobulin E (IgE)-mediated pathology.^8^^,^^9^ IgE-mediated FAs are characterized by type I hypersensitivity reactions.^10^ The IgE-driven mechanism is the most common cause of clinical conditions summarized under the term “food allergy” and is different from nonimmunogenic food intolerance (such as lactose intolerance), food poisoning, or pharmacological responses to food components (such as caffeine and glutamates).^1^^,^^11^, ^12^, ^13^ IgE-mediated FA affects an estimated 3%–10% of adults and 8% of children worldwide.^14^ Symptoms can range from mild to severe and can involve multiple organ systems.^10^ Aside from the risk of severe and even fatal consequences of FA, there is considerable evidence that FA poses a strong negative impact on health-related quality of life (HRQoL) including social, emotional, and physical functioning, and psychological burden (with greater symptoms of anxiety and depression) to patients and their families/caregivers. In addition, FA incurs a substantial socioeconomic burden owing to direct and indirect costs, not only to the patients and their families but also to the healthcare system, taxpayers and society. It is also worth observing that the total socioeconomic burden and total costs due to FA varies by region/country, and the kind of FA.^10^^,^^15^, ^16^, ^17^

Current management strategies for FA focus primarily on allergen avoidance and symptom management.^12^ Allergen avoidance is the simplest form of management but is often not successful over the long term due to accidental exposures.^12^^,^^18^ A retrospective study in peanut-allergic children found accidental peanut ingestion rates of 14% per year.^19^ In a separate longitudinal observational study, approximately 12% of children with peanut allergy experienced adverse reactions from accidental peanut exposure despite best efforts at allergen avoidance.^20^ According to the European Academy of Allergy and Clinical Immunology (EAACI), recommendations for management of FA include the use of epinephrine/adrenaline in patients with risk of anaphylaxis and acute life-threatening symptoms while antihistamines may have value for the treatment of acute non-life-threatening symptoms.^12^

Oral immunotherapy (OIT) has also been shown to be effective in the management of FA. OIT is based on the administration of increasing doses of the allergenic food until a maintenance dose is reached, at which point many patients continue regular ingestion to maintain desensitization to prevent reactions from accidental ingestions. However, OIT carries significant risks; allergic reactions to accidental food allergen exposure may not be completely eliminated and allergic reactions to maintenance dosing may occur, and long-term need for continued maintenance dosing in most is required to sustain desensitization.^21^, ^22^, ^23^ Palforzia, a therapeutic peanut allergen powder, is currently the only approved (United States Food and Drug Administration and European Medicines Agency approved^24^^,^^25^) OIT product and is used for the treatment of peanut allergy in children.^21^^,^^26^ However, many patients are sensitized to multiple allergens, and would therefore require broader-spectrum OIT. Eosinophilic gastrointestinal (GI) disease and serious adverse reactions including anaphylaxis also need to be balanced with the potential benefits of OIT.^27^

There is an urgent requirement to address the unmet needs of patients with FA, particularly of those with multiple FAs as they are more likely to experience accidental exposure and often need to avoid several food groups, which can lead to harmful effects on nutrition, growth, and quality of life (QoL).^28^ Ligelizumab is currently under investigation for the management of IgE mediated FA. This article will provide the rationale for development of ligelizumab in FA based on its unique mechanistic characteristics and its existing efficacy and safety data in other therapeutic areas. In this context, this article will provide an overview of the central role of IgE in FA and the existing evidence for anti-IgE therapies in FA.

## The IgE/FcεRI pathway is considered a major disease driver in FOOD ALLERGY

### Initial allergen sensitization

The development of FA begins with a period of sensitization that may occur through multiple pathways, often including early cutaneous exposure to food protein through a compromised skin barrier. Langerhans cells in the epidermis may take up these food proteins and induce a type 2 helper T cell (Th2) response and IgE production by B cells.^29^^,^^30^ In addition, allergenic foods ingested in the presence of damaged gut epithelia may also produce inflammatory cytokines—the so-called alarmins—that include interleukin (IL)-25, IL-33, and thymic stromal lymphopoietin. These factors activate type 2 innate lymphoid cells, mast cells, and dendritic cells. In a pro-inflammatory cytokine environment, dendritic cells take up and process the antigen to smaller peptides for antigen presentation to naïve T cells via the major histocompatibility complex. This interaction occurs in tissues and lymph nodes and leads to the differentiation of naïve T cells into Th2 cells. These cells produce various cytokines, including IL-4, IL-5, and IL-13, thereby promoting gut infiltration of eosinophils and basophils leading to downstream target effects that promote allergic sensitization and inflammation. In addition, T cell–derived IL-4 and IL-13 drive isotype class switching and B-cell differentiation into plasma cells, producing food allergen–specific IgE antibodies—a process that can occur in classical lymphoid organs and locally in the GI tissues. IgE synthesized in the mucosa is transported through the epithelium into the gut lumen, where they capture allergens that are delivered back to the mucosa.^2^^,^^31^

IgE binds via its fragment crystallizable (Fc) domain to its two major receptors, the high-affinity IgE receptor (FcεRI) and the low-affinity IgE receptor (FcεRII) or CD23 (hereafter always referred to as CD23).^32^ FcεRI is mainly expressed on mast cells, basophils, dendritic cells, intestinal epithelium, and airway smooth muscle cells, whereas CD23 is found on B cells, monocytes, epithelial cells, airway smooth muscle cells, eosinophils, and platelets. Accordingly, allergens can elicit a wide variety of effects upon crosslinking of receptor-bound IgE depending on the receptor and cell type.^33^^,^^34^

### Secondary allergen challenge

**Fig. 1:**
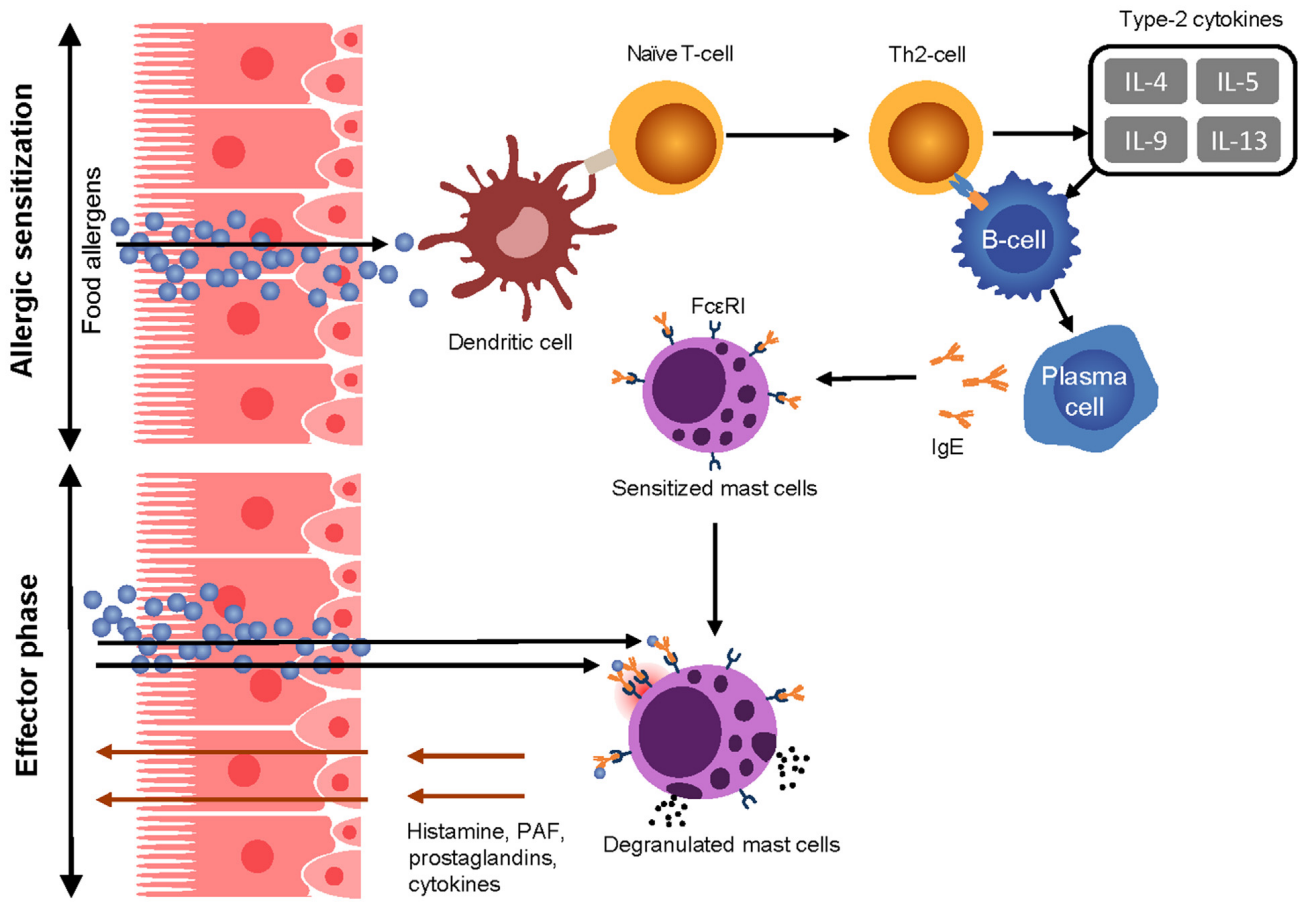
Key pathogenic aspects of IgE-mediated food allergy, After food intake, food proteins are processed by antigen presenting cells of the gut and presented to the naïve CD4 T cells. These CD4 T cells differentiate into Th2 cells and produce type-2 cytokines (IL-4, IL-5, IL-9, and IL-13), promoting the differentiation of B cells into IgE-producing plasma cells. Re-exposure to food allergen leads to cross-linking of allergen-specific IgE bound to FcεRI on mast cells, inducing degranulation and release of several allergic mediators.,^49^ FcεRI, high-affinity IgE receptor; gE, immunoglobulin E; IL, interleukin; PAF, platelet-activating factor

Re-exposure to the allergen, most often through oral ingestion, leads to the crosslinking of FcεRI-bound IgE on mast cells and basophils resulting in cell degranulation and release of vasoactive and pro-inflammatory mediators.^35^^,^^36^ This immediate hypersensitivity reaction plays an important role in the acute allergic response and is mediated by the release of preformed mediators such as histamine, tryptase, and chymase. In addition, allergen crosslinking of IgE/FcεRI activates *de novo* synthesis of leukotrienes, prostaglandins, and platelet-activating factor that contribute to the characteristic symptoms of an allergic reaction and anaphylaxis.^37^, ^38^, ^39^, ^40^ In addition, IgE may be involved in sustaining the type 2 immune response in several different ways. Allergen-induced crosslinking of IgE bound to FcεRI on plasmacytoid dendritic cells (pDC) impairs the natural function of these cells to promote development of regulatory T cells. This process could contribute to unrestricted development of Th2 cell–biased immune response found in food-allergic patients. Furthermore, IgE–allergen complexes, via binding to FcεRI or CD23, can promote antigen presentation by a variety of antigen-presenting cells with subsequent release of type 2 cytokines by polarized Th2 cells.^37^, ^38^, ^39^, ^40^, ^41^, ^42^, ^43^, ^44^ IgE also increases the sensitivity of FcεRI-expressing cells to IgE-mediated responses by stabilizing and thereby enhancing the expression of FcεRI.^45^, ^46^, ^47^ Finally, IgE can actively transport food allergens into the gut mucosa in the form of immune complexes via transcytosis through gut epithelial cells.^48^ [Fig. 1].

## Existing evidence for anti-IgE therapy in FOOD ALLERGY

As IgE plays a significant role in the pathology of FA, the investigation of an anti-IgE therapy for FA is well reasoned.^50^, ^51^, ^52^, ^53^, ^54^, ^55^, ^56^, ^57^, ^58^, ^59^, ^60^, ^61^, ^62^, ^63^ TNX-901 (talizumab) was the first anti-IgE to be studied in the treatment of FA and was shown to protect most patients against allergic reactions from accidental peanut exposure by substantially increasing the peanut reaction threshold.^50^ Once-monthly subcutaneous talizumab 150–450 mg demonstrated a dose-dependent increase in the threshold of peanut to induce a clinical reaction. The efficacy of talizumab 450 mg was statistically significant vs placebo (*p* < 0.001), with subjects showing an average ∼16-fold increase in their peanut reaction threshold.^50^ However, talizumab was not developed further in FA.

Omalizumab is the only monoclonal anti-IgE antibody approved for clinical use, currently for the treatment of severe allergic asthma, CSU, and severe nasal polyposis. It has also been investigated for the treatment of FA, both as monotherapy and in combination with OIT. Studies involving omalizumab have evaluated treatment for peanut, milk, and multi-food allergies, and all have produced encouraging results.^51^, ^52^, ^53^, ^54^^,^^64^^,^^65^

In an unblinded study, marked changes in peanut challenge thresholds were observed after only 1–3 doses of omalizumab.^66^ In a double-blind, placebo-controlled trial, in combination with milk OIT, long-term treatment with omalizumab helped reduce the number of dose-related adverse reactions of OIT. Following 28 months of treatment, desensitization was seen in 88.9% of patients with omalizumab add-on treatment to OIT compared with 71.4% of patients with OIT alone (*p* = 0.18). Sustained unresponsiveness to oral food challenge by month 32 demonstrated that ∼48.0% of patients maintained desensitization in the omalizumab add-on group vs 35.7% in the placebo group (*p* = 0.42).^57^ Similar results were observed in a randomized, double-blind, placebo-controlled study that investigated the effect of omalizumab on sustained desensitization to peanut OIT.^59^ In a randomized, double-blind, placebo-controlled trial that investigated desensitization with multi-food OIT in combination with omalizumab, the combination of omalizumab and OIT significantly improved desensitization to multiple trigger foods in patients with multiple FAs, with a significantly greater proportion of patients passing the double-blind, placebo-controlled food challenge vs OIT only (83% vs 33%; *p* = 0.004).^61^ More recently, the effect of sustained unresponsiveness with omalizumab-facilitated multi-allergen OIT has been explored in a randomized, double-blind, placebo-controlled Phase 2 pilot trial. The results showed that sustained desensitization after omalizumab-facilitated multi-OIT is higher through continued maintenance OIT dosing of either 300 mg or 1 g of each food allergen as opposed to discontinuation of multi-allergen OIT (85% vs 55%, *p* = 0.03).^62^ Rates of sustained unresponsiveness following omalizumab-facilitated multi-OIT appeared higher than those achievable with OIT alone; however, larger studies are needed to explore this further. Omalizumab in FA is currently being investigated in an ongoing Phase 3 study (OUtMATCH: Omalizumab as Monotherapy and as Adjunct Therapy to Multi-Allergen OIT in Food Allergic Participants) (NCT03881696).

## Next generation anti-IgE ligelizumab — key characteristics

**Fig. 2:**
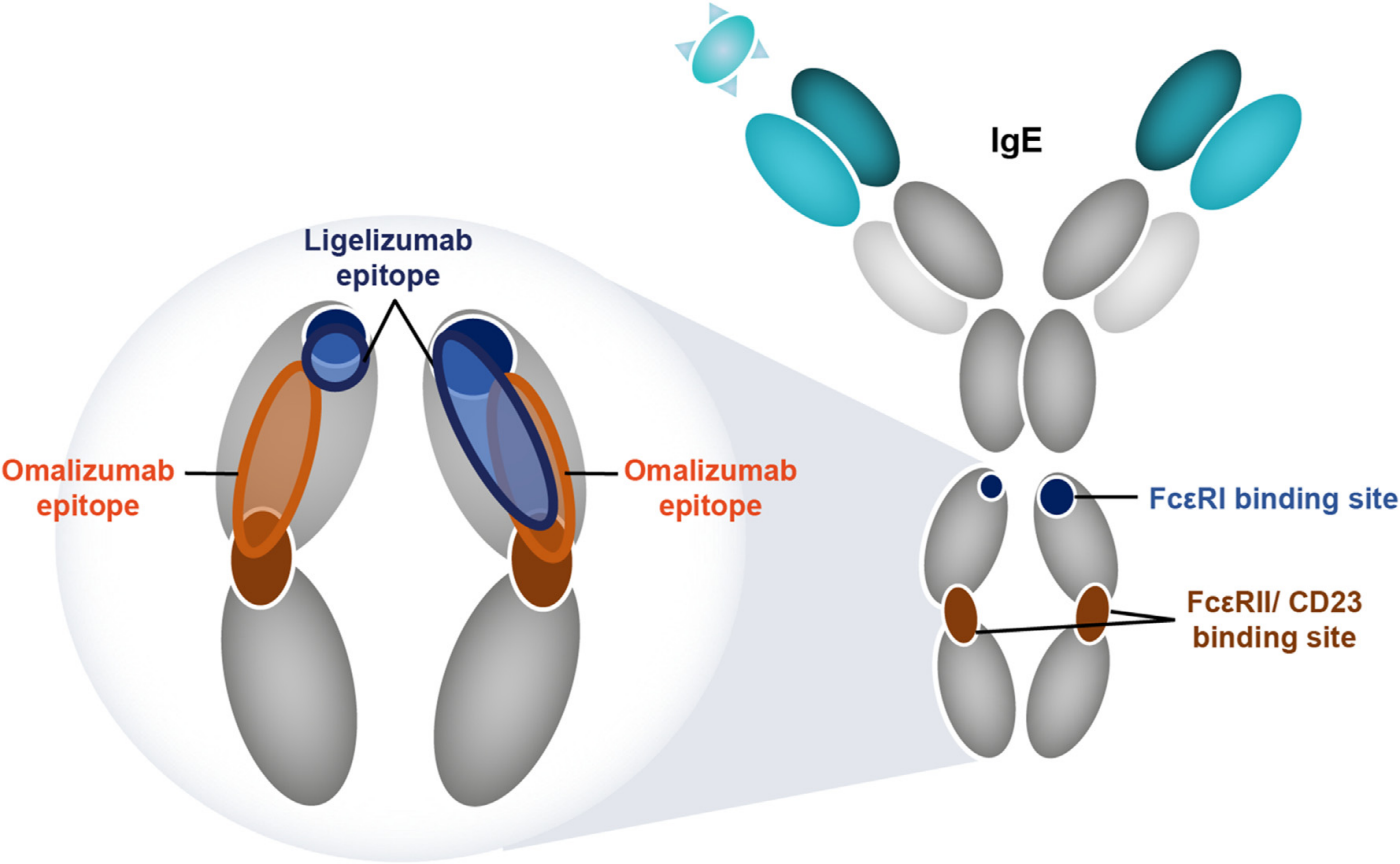
Binding sites of omalizumab and ligelizumab on IgE: The two anti-IgE antibodies recognize similar but distinct epitopes on IgE. The ligelizumab epitope significantly overlaps with the binding site of FcεRI receptor and has only minor overlap with the CD23 receptor. The epitope of omalizumab is located more closely to the binding site of CD23. Consequently, ligelizumab more potently inhibits IgE binding to FcεRI than omalizumab, whereas omalizumab blocks IgE binding to CD23 more potently than ligelizumab.,^67^^,^^68^ FcεRI, high-affinity IgE receptor; FcεRII/CD23, low-affinity IgE receptor; IgE, immunoglobulin E

**Fig. 3:**
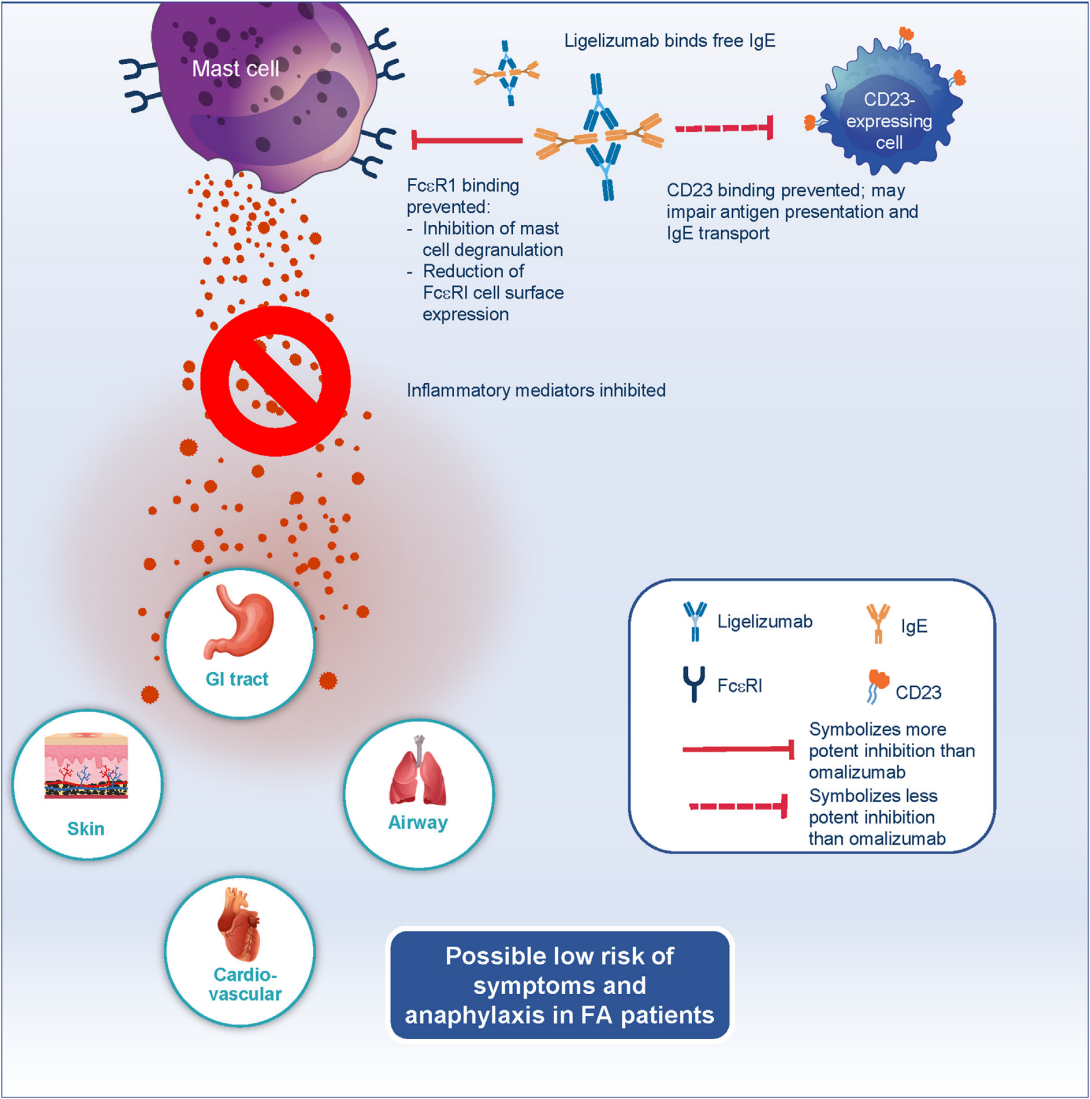
Consequences of ligelizumab-mediated IgE binding blockade to FcεRI and CD23 expressing cells. Ligelizumab binds to free IgE and blocks its binding to FcεRI expressed on mast cells and basophils. The reduced availability of free IgE leads to the loss of FcεRI receptor numbers, which contributes to the effects of ligelizumab. The combined effects lead to a reduction in allergen-induced activation of mast cells and basophils, thereby reducing the release of pro-inflammatory mediators, with the potential to prevent food-induced allergic response and anaphylaxis.^67^ Ligelizumab also blocks the IgE/CD23 pathway, which may have implications for antigen presentation and IgE transport.,^72^^,^^73^ GI, gastrointestinal tract; FA, food allergy, FcεRI, high-affinity IgE receptor; CD23, low-affinity IgE receptor; IgE, immunoglobulin E

Ligelizumab, a derivative of talizumab, is a humanized immunoglobulin-G1 (IgG1/κ) monoclonal antibody that binds to human IgE with ∼88-fold higher affinity than omalizumab, as observed through *in vitro* laboratory investigation. Ligelizumab recognizes an epitope that spans across the Fc domain Cε3 regions of human IgE and thereby blocks IgE binding to FcεRI and CD23.^67^^,^^68^ The epitope of ligelizumab on IgE is different from that recognized by omalizumab and shows greater overlap with the binding site of FcεRI compared with omalizumab (Fig. 2). The different epitopes recognized by the two anti-IgE antibodies translate into a qualitatively different IgE inhibition profile whereby ligelizumab inhibits the binding of free IgE to FcεRI more potently than omalizumab. Conversely, omalizumab is more potent in blocking the binding of IgE to CD23 than ligelizumab. Consequently, ligelizumab displays higher potency in the blocking of IgE/FcεRI signaling resulting in a strong reduction in the inflammatory mediator release from the mast cells (Fig. 3). In patients treated with ligelizumab, circulating IgE is rapidly neutralized and becomes inaccessible to IgE receptors, thereby blocking the crosslinking of receptor-bound IgE and its downstream effects as evidenced by inhibition of skin prick test responses.^69^^,^^70^ As already discussed, the IgE/FcεRI pathway is considered a major disease driver in FA;^71^ therefore, ligelizumab is viewed as a promising candidate molecule to be investigated in the management of patients with FA.

## Available clinical evidence on the efficacy of ligelizumab in asthma and CHRONIC SPONTANEOUS URTICARIA

Details of completed and ongoing ligelizumab clinical trials in asthma and CSU are listed in Table 1.

**Table 1:** Completed and ongoing clinical trials of ligelizumab in asthma and CSU^a^

NCT number	Study Phase	Number of patients/planned/actual enrolment	Study population	Study description	Recruitment Status (Study Start date–End date)
NCT01703312	Phase 2	37	Asthma	Randomized, double-blind, placebo- and comparator-controlled study evaluating the effect of multiple doses of ligelizumab compared with omalizumab in asthma induced by allergen bronchial provocation	Completed (Nov 2012–Oct 2013)
NCT01716754	Phase 2	471	Asthma	Multicenter, randomized, double-blind, placebo- and active-controlled study with exploratory dose-ranging to investigate the efficacy and safety of 16-week treatment with subcutaneous ligelizumab in asthma patients not adequately controlled with high-dose inhaled corticosteroids and long-acting β_2_-agonists	Completed (Dec 2012–Jan 2016)
NCT02477332	Phase 2b	382	CSU	Multicenter, randomized, double-blind, placebo- and active-controlled dose-finding study of ligelizumab as an add-on therapy to investigate the efficacy and safety in patients with CSU	Completed (Jul 2015–Jun 2017)
NCT02649218	Phase 2	226	CSU	Open-label, multicenter, extension study to evaluate the long-term safety of ligelizumab 240 mg s.c. given every 4 weeks for 52 weeks in CSU patients who completed study NCT02477332	Completed (May 2016–May 2019)
NCT03437278	Phase 2b	49	CSU	Dose-finding, multicenter, randomized, double-blind, placebo-controlled, parallel-group study to investigate the efficacy and safety of ligelizumab in adolescent patients with CSU	Completed (Aug 2018–Feb 2021)
NCT03580369	Phase 3	1072	CSU	Multicenter, randomized, double-blind, active- and placebo-controlled, parallel-group study to investigate the efficacy and safety of ligelizumab in the treatment of CSU in adolescents and adults inadequately controlled with H_1_-antihistamines	Completed (Oct 2018-Jun 2022)
NCT03580356	Phase 3	1079	CSU	Multicenter, randomized, double-blind, active- and placebo-controlled, parallel-group study to investigate the efficacy and safety of ligelizumab in the treatment of CSU in adolescents and adults inadequately controlled with H_1_-antihistamines	Completed (Oct 2018-Jun 2022)
NCT03907878	Phase 3	66	CSU	Multicenter, open-label study to investigate the safety/tolerability and efficacy of ligelizumab in the treatment of adult Japanese patients with CSU inadequately controlled with H_1_-antihistamines	Completed (Apr 2019–Jan 2022)
NCT04210843	Phase 3	1038	CSU	Multicentre, double-blinded and open-label extension study to evaluate the efficacy and safety of ligelizumab as retreatment, self-administered therapy, and monotherapy in CSU patients who completed previous studies of ligelizumab in CSU	Active – not recruiting (Apr 2020–Ongoing)
NCT04513548	Phase 1	11	Part 1: Healthy subjects and CSU patients Part 2: Chronic urticaria patients (CSU, cholinergic urticaria or cold urticaria)	A two-part, randomized double-blind study to investigate the MechAniSm of acTion of ligElizumab treatment in patients with chronic uRticaria (MASTER)	Terminated (Company decision)

CSU, chronic spontaneous urticaria; NCT, National Clinical Trial; s.c., subcutaneous.

a Details of the studies including the recruitment status is as of August 2022 (https://clinicaltrials.gov/)

Two Phase 2 clinical trials have investigated ligelizumab in allergic asthma following one Phase 1 trial in atopic subjects. In a Phase 1 trial, ligelizumab showed greater reduction in skin prick wheal allergic responses in atopic subjects compared with omalizumab (>95% vs 41%; *p* < 0.001). A dose-dependent reduction in circulating free IgE levels and basophil cell surface–bound IgE levels was also observed with ligelizumab. In addition, ligelizumab demonstrated a dose- and time-dependent reduction in basophil FcεRI and IgE levels.^69^ In a Phase 2 trial in patients with mild allergic asthma, ligelizumab at a dose of up to 240 mg once every 2 weeks (Q2W) showed superior suppression of the skin prick test (*p* = 0.002 and *p* < 0.0001 for 72 mg and 240 mg, respectively) and a ∼3-fold greater reduction in reactivity (bronchial airway allergen PC_15_ response) to the allergen compared with omalizumab (dosed as per the dosing table posology^65^) at Week 12 (240 mg; *p* = 0.10).^70^ However, a ligelizumab Phase 2 study in patients with severe allergic asthma did not achieve the primary endpoint of ≥0.5-point difference in Asthma Control Questionnaire-7 score to demonstrate superiority for ligelizumab vs placebo at Week 16.^74^ This asthma study provided first insights that ligelizumab and omalizumab may have different properties, which may result in different clinical outcomes in a highly complex disease such as asthma.^74^ Indeed, a subsequent pre-clinical study determined significant differences in molecular binding profile and functional modes-of-action of ligelizumab compared with omalizumab.^67^

With regard to CSU, a Phase 2b study in adult patients demonstrated a dose-dependent response to ligelizumab with more patients achieving complete control of disease activity compared with omalizumab and placebo at Week 12 (weekly Hives Severity Score = 0 response rates at Week 12 were 30%, 51%, and 42% for ligelizumab 24, 72, and 240 mg, respectively, vs 26% for omalizumab [CSU dose 300 mg] and 0% for placebo).^75^ Patients who completed this Phase 2b study and presented with active disease entered into an open-label safety extension study (NCT02649218). The results showed that one-year treatment with ligelizumab 240 mg Q4W showed no newly identified or unexpected safety concerns. A Phase 2b study (NCT03437278) with adolescent patients with CSU has recently been completed. Ligelizumab received breakthrough designation from the US FDA for CSU and is currently in Phase 3 trials (NCT03580369, NCT03580356, NCT03907878, NCT04210843) for the treatment of CSU in adults and adolescent patients aged ≥12 years. Combined, these studies recruited over 2000 patients to investigate the efficacy and safety of ligelizumab in patients with CSU inadequately controlled by H_1_-antihistamines. At the time of the interim analysis, the 2 identically designed Phase 3 studies (NCT03580369 and NCT03580356) met their primary endpoint of superiority vs placebo (change from baseline in weekly Urticaria Activity Score) at Week 12 in the treatment of CSU; however, superiority vs omalizumab was not demonstrated.^76^

## Ligelizumab – existing evidence on safety and tolerability

In a Phase 1 study in patients with atopy, the most commonly reported adverse events (AEs) were headache and upper respiratory tract infection. Mild-to-moderate urticaria was reported as the most significant AE in this study, with an incidence of ∼17% in any ligelizumab dose group; the event resolved spontaneously or with the use of antihistamines. Only 2 (5%) patients on ligelizumab discontinued due to AEs of asthma exacerbations and flu-like illness, but neither were considered to be related to the study drug.^69^ In the Phase 2/2b studies in patients with mild allergic asthma (NCT01703312), severe uncontrolled asthma (NCT01716754 [core study]) and NCT02075008 [extension study], and CSU (NCT02477332 [core study]) and NCT02649218 [extension study]), the most common AEs (occurring in ≥10% of patients) reported in any ligelizumab group were nasopharyngitis, asthma, oropharyngeal pain, diarrhea, headache, injection site reaction, injection site erythema, nasal congestion, viral upper respiratory tract infection, urinary tract infection, and urticaria.^70^^,^^75^^,^^77^ No ligelizumab dose-response relationship was observed in the occurrence of these events. No patient with mild allergic asthma discontinued the study due to AEs,^70^ while a very small proportion of patients with CSU (∼2%) on any ligelizumab dose discontinued due to AEs, which was similar to or fewer than those discontinuing in the omalizumab and placebo arms (2% and 5%, respectively).^75^ In general, across these Phase 1 and Phase 2 studies, a majority of the AEs were mild to moderate in severity and were not suspected to be related to the study drug, with the exception of mild-to-moderate injection-site reactions. Serious AEs were reported in the Phase 2b core and extension studies in patients with CSU^75^^,^^77^ and in the Phase 2 core (NCT01716754) and extension studies (NCT02075008) in patients with asthma. In CSU Phase 2b core study, 11 (4.3%) patients on any ligelizumab dose experienced serious AEs, which was similar to that observed in the omalizumab group (3 [4%]) and lower than that in the placebo group (4 [9%]).^75^ In the CSU Phase 2b extension study, overall, 26 treatment-emergent serious AEs were reported in a total of 15 patients (6.6%) on ligelizumab, of which only the event of hypersensitivity (one case; [0.4%]) was reported to be related to the study drug, and this event has been positively adjudicated as anaphylaxis.^77^ In the asthma core study (NCT01716754), 12 (5.0%) patients on any ligelizumab dose, 1 (0.8%) patient on omalizumab, and 5 (5.2%) patients on placebo group experienced serious AEs. Of these, the serious AE of drug hypersensitivity was noted in 1 (0.5%) patient on any ligelizumab dose group. In the asthma extension study (NCT02075008), overall, 19 serious AEs were reported in a total of 270 patients (7.0%) on ligelizumab, that included 1 serious AE (0.4%) of anaphylactic reaction and 1 serious AE (0.4%) of drug hypersensitivity. No clinically meaningful changes in hematology, blood chemistry, urinalysis, vital signs, and electrocardiographic recordings have been reported with ligelizumab. No deaths in ligelizumab treatment arms have been reported in any Phase 1 or Phase 2 studies. Overall, ligelizumab has been reported to be well tolerated with doses ranging from 12 mg once every 2 weeks (Q2W) to 240 mg Q2W.^69^^,^^70^^,^^74^^,^^75^^,^^77^

## Prospect for ligelizumab in FOOD ALLERGY

In early Phase 1 trials, ligelizumab showed dose- and time-dependent suppression of allergen-elicited wheals with a standardized skin prick test.^69^^,^^70^ Given that the skin prick test is one of the recommended diagnostic tools for various allergic diseases such as FA, this effect of ligelizumab in reducing the skin prick wheal allergic response and free IgE levels may translate into beneficial effects in such diseases.^78^ Based on previous ligelizumab studies and extrapolation to patients with FA, modeling of the pharmacokinetic (PK)/pharmacodynamic (PD) basophil FcεRI show that the selected dose regimens of 120 mg and 240 mg every 4 weeks (Q4W) may be most appropriate for the majority of patients with FA.^79^ The ongoing Phase 3 study of ligelizumab in FA is a randomized, double-blind, placebo-controlled, 52-week study assessing 2 dose regimens (120 mg and 240 mg administered subcutaneously Q4W) in patients aged 6–55 years with a confirmed diagnosis of peanut allergy (NCT04984876). The reason for selecting the same dose range for patients in different age groups is based on the fact that pediatric patients starting 6 years have higher baseline IgE levels than adults.^80^ As with omalizumab, dosing will be restricted in patients with elevated IgE levels with an upper limit of 2000 IU/mL in the planned Phase 3 trial, a study that will provide more clarity on the efficacy and safety of ligelizumab in patients with FA and provide guidance on the optimal posology.

## Conclusions

Although the financial and psychosocial burden of FA is high, and the prevalence of FA in many countries is on the rise, existing treatment options for FA are extremely limited. Due to the important role played by IgE/FcεRI pathway in FA, IgE suppression with monoclonal antibodies, such as talizumab and omalizumab, has been shown to provide clinical benefits in patients with FA. Allergen avoidance and patient nutrition care continues to be a key approach for food allergy management. In addition, anti-IgE therapy has the potential to complement the standard of care by diminishing the risk to the patient. Ligelizumab (a talizumab derivative), is a next generation humanized monoclonal antibody that shows greater overlap with the binding site of FcεRI and binds to human IgE with ⁓88-fold higher affinity compared with omalizumab. Accordingly, ligelizumab has displayed high potency to block IgE/FcεRI signaling in both *in vitro* and *in vivo* studies. Consequently, considering its potent suppression of both serum IgE and skin prick test, and known safety profile, ligelizumab is a promising candidate to be investigated in the management of patients with IgE-mediated FA.

## Abbreviations

AE, adverse event; CSU, chronic spontaneous urticaria; EAACI, European Academy of Allergy and Clinical Immunology; FA, food allergy; Fc, fragment crystallizable; FcεRI, high-affinity IgE receptor; FcεRII, low-affinity IgE receptor; GI, gastrointestinal; HRQoL, health-related quality of life; IgE, immunoglobulin E; IL, interleukin; OIT, oral immunotherapy; PD, pharmacodynamic; pDC, plasmacytoid dendritic cells; PK, pharmacokinetic; Q2W, once every 2 weeks; Q4W, once every 4 weeks; QoL, quality of life; Th1, type 1 helper T cell; Th2, type 2 helper T cell.

## Acknowledgments

The authors thank Mohammad Fahad Haroon (PhD), Sahaja Banda, and Anupama Boddupalli of 10.13039/100004336Novartis Healthcare, Hyderabad, India, for providing medical writing support which was funded by Novartis Pharma AG, Switzerland in accordance with Good Publication Practice (GPP3) guidelines (http://www.ismpp.org/gpp3).

## Funding

Not applicable.

## Author contributions

All authors made substantial contributions to the conception and design of this article; took part in drafting the article or revising it critically for important intellectual content; agreed to submit to the current journal; gave final approval for the version to be published; and agree to be accountable for all aspects of the work.

## Ethics approval

Not applicable.

## Submission declaration

This manuscript is being submitted only to *World Allergy Organization Journal* and has not been previously submitted to another journal, nor is it currently under consideration by another journal.

## Consent for publication

The authors' consented to the publication of this review.

## Declaration of competing interest

**Robert A Wood** receives research support from NIAID, Aimmune, Astellas, DBV, FARE, Genentech, HAL-Allergy, Novartis, and Regeneron.

**R. Sharon Chinthrajah** reports grants from NIAID, CoFAR, Aimmune, DBV Technologies, Astellas, Regeneron, FARE, and MCHRI; is an advisory member for Alladapt Therapeutics, Genentech, Allergenis, Novartis, Sanofi, and Nutricia; and received personal fees from Before Brands, all outside the submitted work.

**Alexander Eggel** reports consulting activities for Novartis and received research and travel grant support from Novartis.

**Ivan Bottoli, Paolo Tassinari, Aurelie Gautier, and Maximilian Woisetschlaeger** are employees of Novartis Pharma AG, Basel, Switzerland.

**Pablo Altman** is an employee of Novartis Pharmaceuticals Corporation, East Hanover, New Jersey, United States.
